# Toll-like receptors in the brain of mice following infection with *Acanthamoeba* spp.

**DOI:** 10.1007/s00436-016-5217-9

**Published:** 2016-08-11

**Authors:** Agnieszka Wojtkowiak-Giera, Monika Derda, Agnieszka Kolasa-Wołosiuk, Edward Hadaś, Danuta Kosik-Bogacka, Piotr Solarczyk, Paweł P. Jagodziński, Elżbieta Wandurska-Nowak

**Affiliations:** 1 Department of Biology and Medical Parasitology, Poznan University of Medical Sciences, 10 Fredry Street, 61-701 Poznan, Poland; 2 Department of Histology and Embryology, Pomeranian Medical University, 71 Powstancow Wielkopolskich Street, 70-111 Szczecin, Poland; 3 Department of Biology and Medical Parasitology, Pomeranian Medical University, 72 Powstancow Wielkopolskich Street, 70-111 Szczecin, Poland; 4 Department of Biochemistry and Molecular Biology, Poznan University of Medical Sciences, 6 Swiecickiego Street, 60-781 Poznań, Poland

**Keywords:** TLR2, TLR4, Toll-like receptors, Q-PCR, Immunohistochemistry, *Acanthamoeba* spp., Brain, Mouse

## Abstract

The Toll-like receptors (TLRs) of the innate immune system play an important role in the recognition of pathogens such as bacteria, viruses, fungi, and parasites. In this study, we examined the changes in the level of expression of TLR2 and TLR4 mRNA and protein in the brains of mice infected with *Acanthamoeba* spp. The *Acanthamoeba* strains were isolated from a patient with *Acanthamoeba* keratitis (AK) (Ac55) and Malta Lake (Ac43). In the brain isolated from mice at 2 days post-infection (dpi) with *Acanthamoeba* strains Ac55 and Ac43, mRNAs for TLR2 and TLR4 were significantly more strongly expressed in comparison with the uninfected mice. In *Acanthamoeba-*infected mice, TLR2 and TLR4 expression was detected in neurons, glial cells, and endothelial cells within the neocortex. These receptors showed more intense expression in ependymocytes of the choroid plexus of infected mice at 2 dpi. Increased levels of TLR2 and TLR4 mRNA expression in infected mice suggest the involvement of these TLRs in the recognition of *Acanthamoeba* spp. pathogen-associated molecular patterns (PAMPs).

## Introduction

*Acanthamoeba* spp. are free-living amoebae (FLA) found in several natural habitats, including lakes, rivers, swimming pools, thermal baths, tap water, sewage, humid soils, and dust (Khan, 2006).

Traditional taxonomy of *Acanthamoeba* has used morphological characteristics of cysts and trophozoites (Booton et al. 2005). However, genetic studies have led to the identification of 18 genotypes (T1–T18) based on rRNA gene sequences (Qvarnstrom, et al. 2013). The T4 genotype has been frequently reported as a predominant cause of AK (Niyyati et al. 2009).

*Acanthamoeba* spp. can infect humans and animals as opportunistic pathogens and cause severe diseases, including amebic *Acanthamoeba* keratitis (AK), a painful sight-threatening infection of the cornea, and granulomatous amebic encephalitis (GAE), a fatal disease of the central nervous system (CNS), in immunocompromised hosts (Martinez & Visvesvara, 1997; Visvesvara et al. 2007). The important clinical symptoms of GAE are headache, fever, behavioral changes, lethargy, stiff neck, aphasia, ataxia, nausea, cranial nerve palsies, confused state, seizures, and coma, which finally lead to death. Pathological findings include hemorrhagic necrosis, fibrin thrombi, and inflammation (Marciano-Cabral & Cabral 2003). The intensity of symptoms and histological changes in the host may be a result of many factors including the immunocompetence of the host and the virulence of amoebae. Experimental studies have shown that the properties of pathogenic free-living amoebae and the intensity of histological changes in organs depend on the virulence of the strain (Rucka, 1974) and, on the other hand, the duration of infection (Gieryng et al. 1993). In mice infected with different strains of *Acanthamoeba* spp., Górnik et al. (2005) demonstrated that the changes in intensity in the brain depend on the virulence of the strain.

*Acanthamoeba* spp. infections of the skin, nasal passages, lung, and brain are also documented in patients with immunodeficiency disease (Martinez & Visvesvara 1997; Marciano-Cabral & Cabral, 2003). Furthermore, several studies strongly suggest that *Acanthamoeba* spp. can act as reservoir hosts for other pathogenic viruses, bacteria, and fungi (Barker & Brown 1994; Scheid et al. 2008; Gaze et al. 2011; Scheid & Schwarzenberger 2012). In addition, although GAE occurs in healthy people, immunocompromised or debilitated patients due to HIV infection, diabetes, immunosuppressive therapy, malignancies, malnutrition, and alcoholism are particularly at risk (Visvesvara et al. 2007).

The immune defense mechanisms that operate against *Acanthamoeba* have not been well characterized. It was found that in the host defense mechanisms against *Acanthamoeba* spp., both innate and acquired immunities play a role (Cursons et al. 1980; Marciano-Cabral & Cabral 2003). McClellan (2002) found that trophozoites as well as cysts are recognized by the immune system of the host. The innate immunity was the first line of defense against *Acanthamoeba* infection (Ferrante & Rowan-Kelly 1983). Ferrante and Abell (1986) as well as Stewart et al. (1992) demonstrated in vitro killing of trophozoites of *Acanthamoeba* spp. in the presence of neutrophils and macrophages. However, activation in response to infection with *Acanthamoeba* and the role of antibodies are not known (Marciano-Cabral & Cabral 2003). Antibodies may prevent attachment to host cells, inhibit the motility of amoebae, or neutralize ameba cytotoxic factors (Cursons et al. 1980; Ferrante & Abell 1986; Stewart et al. 1994; Marciano-Cabral & Toney 1998).

The pathogenesis of infections by *Acanthamoeba*, including the cellular processes and molecules involved in the recognition and adhesion to the host tissues, is little known. However, Soto-Arredondo et al. (2014) suggested that glycoproteins on the surface of *Acanthamoeba* trophozoites interact with and recognize receptors on the host cell.

The innate immune response in the brain and other tissues is initiated via recognition of pathogen-associated molecular patterns (PAMPS) by pathogen recognition receptors (PRRs) such as the Toll-like receptors (TLRs) (Creagh & O’Neil 2006). To date, 13 TLRs have been identified in mammals, each of which recognizes specific PAMPS or host-derived damage-associated molecular patterns (DAMPS) (Roach et al. 2005; Akira et al. 2006). Signaling via the TLR pathway leads to the production of inflammatory cytokines, chemokines, adhesion molecules, and costimulatory molecules (Ospelt & Gay 2010).

In this study, we examined two selected TLRs: 2 and 4. TLR2 and TLR4 are the best known transmembrane receptors and the most extensively analyzed members of the TLR family. The alteration of TLR2 and TLR4 expression in infected rats indicates the potential role of the innate immune system in the pathomechanism of *Hymenolepis diminuta* infection (Kosik-Bogacka et al. 2012; Kosik-Bogacka et al. 2013). Recent studies have shown that TLR2 is capable of recognizing ligands such as glycosylphosphatidylinositol (GPI) of *Plasmodium falciparum*, *Toxoplasma gondii*, *Trypanosoma cruzi*, *Trypanosoma brucei*, *Leishmania major*, and *Leishmania donovani* (Krishnegowda et al. 2005; Debierre-Grockiego et al. 2007; Chandra & Naik 2008; Egan et al. 2009; Amin et al. 2012). The TLR4 ligands including lipophosphoglycans (LPG) of *Leishmania* spp. (Tuon et al. 2008) and lysophosphatidylserine of *Schistosoma* spp. (van der Kleij et al. 2002; Layland et al. 2007; Van der Kleij et al. 2004) confirm that the phosphatidylserine fraction of *Schistosoma haematobium* contains a TLR2 ligand as well as TLR4. Our previous study confirmed an increase in the level of expression of TLRs 2, 3, 4, and 9 during experimental hymenolepidosis (Kosik-Bogacka et al. 2012, 2013, 2014).

TLRs are predominantly expressed on immune cells but also on non-immune cells. TLR4 is also expressed in the brain cells, in particular parenchymal glial cells, microglia, astrocytes, and in neurons (Rolls et al. 2007; Acosta & Davies 2008; Tu et al. 2011). However, the role of neuronal TLR4 in the central nervous system is unknown (Leow-Dyke et al. 2012). TLR2 is an important element of the brain innate immune response system. TLR2 is also expressed on microglia, astrocytes, neurons, and endothelial cells (Laflamme et al. 2001; Bsibsi et al. 2002), and similarly, the functional significance of this receptor is still unknown (Kielian et al. 2005). Therefore, the aim of this study was to characterize for the first time the expression of TLR2 and TLR4 in the brain of *Acanthamoeba* spp.-infected mice using quantitative real-time polymerase chain reaction (Q-PCR) and immunohistochemical staining (IHC). The *Acanthamoeba* spp. were isolated from a patient with AK (Ac55) and Malta Lake (Ac43).

## Materials and methods

### *Acanthamoeba* spp.

The amoebae isolated from a patient with AK (strain Ac55) and from environmental samples of water from Malta Lake in Poznań, Poland (strain Ac43), were cultured on a non-nutrient agar covered by bacteria *Enterobacter aerogenes* at a temperature of 28 °C. After 2–3 days of culture, amoebae were washed and used for infection or research.

### Genotyping of *Acanthamoeba*

The DNA amplification was performed using genus-specific primers previously described by Schroeder et al. (2001). A set of primers that included the forward JDPI (5′GGCCCAGATCGTTTACCGTGAA′3) and the reverse primer JDP2 was used (5′TCTCACAAGCTGCTAGGGAGTCA′3) for genetic characterization targeting an ∼450-bp fragment of the *Acanthamoeba* 18S rRNA gene. Amplification involved use of a 25-μl suspension of the following reagents: 2.5 mM MgCl_2_, 0.6–1 μM of each primer, 0.2 mM of each deoxynucleotide triphosphate, and 0.5 U of AmpliTaq Gold DNA polymerase. A clinical isolate of *A. castellanii* belonging to the T4 genotype isolated from a keratitis patient (ATCC 00000) was used as a positive control. A negative control consisting of a reaction mixture without a DNA template was included. PCR was carried out using a GeneAmp 2400 thermocycler. Two PCR products were cleaned and sequenced in both directions with the same set of primers. Sequencing was performed with BigDye Terminator v3.1 on an ABI Prism 3130XL Analyzer (Applied Biosystems, USA). Trace files were checked and edited using FinchTV 1.3.1 (Geospiza Inc., Seattle, USA). Contigs were aligned and manually assembled in GeneDoc v. 2.7.000 (Nicholas et al. 1997). Next, the gene sequence fragments of the *Acanthamoeba* isolates were compared with the reference sequences deposited in GenBank (National Center for Biotechnology Information).

### Animals

BALB/c mice, 2–3 weeks old, body weight 10–15 g, were bred and housed in our animal laboratory, which ensured approximately constant temperature, humidity, and ad libitum access to standardized granulated food and water. Mice lightly anesthetized were intranasally infected with one drop of suspension containing 2 × 10^4^ amoebae. Control mice were given the same volume of physiological solution. After inoculation, the animals were monitored constantly.

The experimental material consisted of brains from mice infected with two different strains of *Acanthamoeba* isolated from a patient with AK and from environmental samples.

The mice (*n* = 54) were divided into nine groups:

- Control group 0 (*n* = 6)—uninfected, 0 days post-*Acanthamoeba* infection (0 dpi)

The mice infected by *Acanthamoeba* strain Ac55:

- Group I (*n* = 6)—2 dpi
- Group II (*n* = 6)—4 dpi
- Group III (*n* = 6)—16 dpi
- Group IV (*n* = 6)—30 dpi

The mice infected by *Acanthamoeba* strain Ac43:

- Group I′ (*n* = 6)—2 dpi
- Group II′ (*n* = 6)—4 dpi
- Group III′ (*n* = 6)—16 dpi
- Group IV′ (*n* = 6)—30 dpi

The section infected mice with *Acanthamoeba* at 2, 4, 16, and 30 dpi, depending on the symptoms of infection such as lack of mobility, depression, turning in circles, tousled (matted) hair, anorexia, or emaciation (wasting).

The study was approved by the Local Ethics Committee for Scientific Experiments on Animals in Poznań (Poland).

### Evaluation of infection of animals

Fragments of brains were collected from experimental animals at 2, 4, 16, and 30 dpi.

Sterile collected tissues were applied on 1.5 % agar plates covered with a layer of *E. aerogenes*. The agar plates were incubated at 25 °C. Growth of *Acanthamoeba* on agar plates was observed by microscope at ×40–100 magnification. Animals were regarded as infected when the presence of amoeba was identified on the agar.

### Isolation of RNA and conversion of cDNA by reverse transcription

The expression of TLR2 and TLR4 genes at the mRNA level in brains in mice of five groups (control and 2, 4, 16, 30 dpi) was examined using reverse transcription polymerase chain reaction (RT-PCR). The brains were homogenized in liquid nitrogen, and total RNA was isolated according to the manufacturer’s instructions (Qiagen, Germany). One microgram of RNA from segments of lungs was reverse transcribed with an oligo (dT) primer in a 20-μl reaction (first-strand cDNA synthesis using M-MLV RT kit; Invitrogen, CA) to obtain cDNA. Successful cDNA conversions were confirmed by amplification using conventional PCR (GeneAmp PCR System 2400, Applied Biosystems).

### Real-time PCR

The expression of TLR2 and TLR4 genes in fragments of brain was measured by Q-PCR. This method enables both detection and quantification of gene expression at the mRNA level. Q-PCR was carried out in a LightCycler real-time PCR detection system from Roche Diagnostic GmbH (Mannheim, Germany) using SYBR Green I as detection dye, and target cDNA was quantified using a relative quantification method using a calibrator. The calibrator was prepared as a cDNA mix from all samples, and successive dilutions were used to create a standard curve as described in the Relative Quantification Manual, Roche Diagnostics GmbH (Mannheim, Germany). The housekeeping gene PBGD was amplified as the reference gene for mRNA quantification. The quantity of TLR2 and TLR4 transcripts in each sample was standardized by the geometric mean of PBGD transcript level. For amplification, 1 μl of total (10 μl) cDNA solution was added to 5 μl of LightCycler 480 DNA SYBR Green I Master (Roche) as well as primers for TLR2, TLR4, and PBGD. One RNA sample of each preparation was processed without RT reaction to provide a negative control in subsequent PCR series. Primers for TLR2 were forward 5′-AAA GAT GTC GTT CAA GGA GG-3′ and reverse 5′-ATT TGA CGC TTT GTC TGA GG-3′ (product—161 bp); TLR4 forward 5′-TTC TTC TCC TGC CTG ACA CC-3′ and reverse 5′-CTT TGC TGA GTT TCT GAT CCA T-3′ (product—94 bp); and PBGD forward 5′-TGG ACC TAG TGA GTG TGT TG-3′ and reverse 5′-GGT ACA GTT GCC CAT CTT TC 3′ (product—138 bp). Real-time data were collected and analyzed using the Excel program. The amounts of TLR2 and TLR4 mRNA are expressed as the multiplicity of these cDNA concentrations in the calibrator.

### Immunohistochemical staining

Paraffin-embedded sections (3–5 μm) of brains from mice infected with *Acanthamoeba* isolated from patients and from Malta Lake (control and 2, 4, 16, 30 dpi) were immunostained for visualization of TLR2 and TLR4 proteins.

Immunohistochemistry was performed using specific primary rabbit polyclonal antibodies against TLR2 and TLR4 (Santa Cruz Biotechnology, Inc., cat. no. sc-10739 and sc-30002) in a final 1:500 dilution. Firstly, the deparaffinized sections were microwave irradiated in citrate buffer (pH 6.0) to heat induce epitope retrieval. After slow cooling to room temperature, slides were washed in PBS twice for 5 min and then incubated with primary antibodies overnight (4 °C). On the next day, sections were stained with an avidin-biotin-peroxidase system with diaminobenzidine as the chromogen (Rabbit ABC Staining System, Santa Cruz Biotechnology, Inc., cat. no. sc-2018) in conformity with staining procedure instructions included. Sections were washed in distilled H_2_O and counterstained with hematoxylin. For a negative control, specimens were processed in the absence of primary antibodies. Positive staining was defined microscopically by visual identification of brown pigmentation. The IHC-stained sections were examined by light microscope (Leica, DM5000B, Germany).

### Statistical analysis

The obtained results were analyzed statistically using Statistica 6.1 software. Arithmetic mean and standard deviation (SD) were calculated for each of the studied parameters. Two-group testing was performed using Student’s *t* test. A value of *P* < 0.05 was considered statistically significant.

## Results

The macroscopic observation confirmed edema and hyperemia in the brain hemispheres of mice infected with *Acanthamoeba*.

### Genotyping of *Acanthamoeba*

The DNA was isolated from two *Acanthamoeba*-positive samples. Amplicons of the fragment of 18S rRNA gene were obtained from the *Acanthamoeba* Ac43 and Ac55 isolates from the water and corneal scrape, respectively. The results showed that sequences obtained from *Acanthamoeba* Ac43 isolates shared 100 % identity to the sequences from the isolates of *Acanthamoeba* obtained from meadow soil (KF928953), gill tissue (HM363628), air conditioner (GQ397470), and river water (EU273824). The comparison of the sequence at the same molecular marker of the *Acanthamoeba* Ac55 isolate from the human with the sequences deposited in GenBank also showed 100 % identity to the sequences of this gene of the parasite isolated from infected liver of pheasant *Tragopan temminckii* (GQ889265), corneal (KF318460, DQ087297) and contact lens (DQ087296) scrapings, and an environmental sample (EU377583) (Table 1).

**Table 1:** Results of genotyping of *Acanthamoeba* sp. from water and clinical sample

Sampling	Isolate, accession no.	Published sequences in the GenBank			
		Accession no.	Sampling, isolate	Region of origin	References
Malta Lake, Poznan	Ac43, KP120879	KF928953	High altitude meadow soil, *Acanthamoeba* sp., Tib121	China	Geisen et al. (2014)
		HM363628	Gill tissue, rainbow trout, *Acanthamoeba* sp., GERF3	Germany	Dyková et al. (2010)
		GQ397470	Air conditioner water, *Acanthamoeba* sp., AcaVN08	Slovakia	Nagyova et al. (2010)
		EU273824	River water, upstream from a drinking water production plant, *Acanthamoeba* sp., CRIB-22	France	Thomas et al. (2008)
Corneal scrape	Ac55, KP120880	GQ889265	CDCV600, liver of a Temminck’s tragopan, *Acanthamoeba* sp., genotype: T4	USA	Visvesvara et al. (2010)
		KF318460	Corneal surface tissue, *Acanthamoeba* sp., 1 FRC-2013	Brazil	Mafra et al. (2013)
		EU377583	Biofilm, *Acanthamoeba* sp., CRIB53	Switzerland	Corsaro et al. (2009)
		DQ087296	Contact lenses and contact lens case, *Acanthamoeba* sp., S6	France	Yera et al. (2008)
		DQ087297	Corneal scraping, *Acanthamoeba* sp., 222BAL	France	Yera et al. (2007)

The *Acanthamoeba* sequences from the isolates obtained from Malta Lake, Poznan (Ac43) and corneal scraping (Ac55) were deposited in GenBank (NCBI) under accession numbers KP120879 and KP120880, respectively.

### Expression of TLR2 and TLR4 genes

**Fig. 1:**
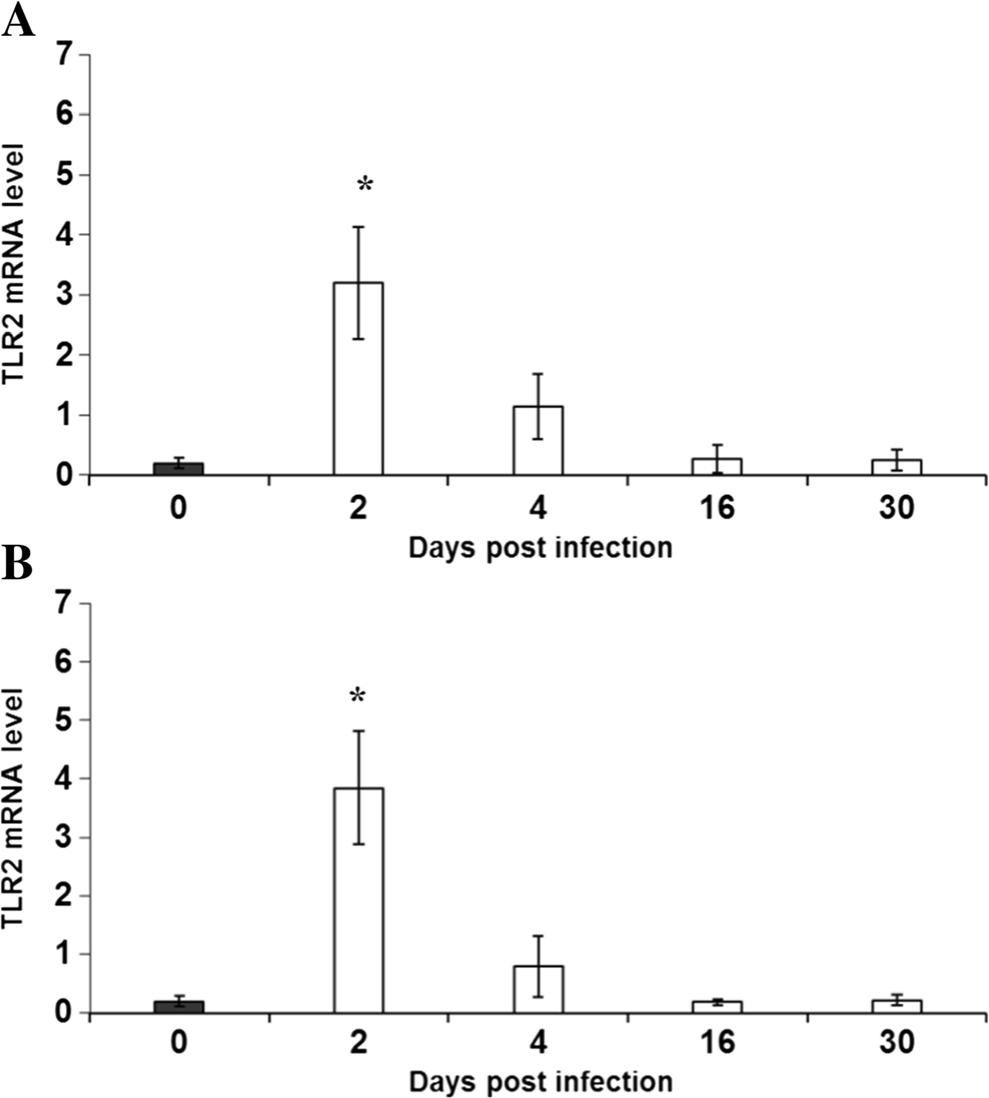
Expression of TLR2 gene at the mRNA level in brains isolated from uninfected and *Acanthamoeba*-infected mice from patient with *Acanthamoeba* keratitis (strain Ac55; **a**) and Malta Lake (strain Ac43; **b**). Brains were dissected from mice at 2, 4, 16, and 30 dpi. Expression level of TLR2 gene was determined by Q-PCR relative quantification analysis evaluated using a calibrator (cDNA mix from all samples). The quantify of TLR2 transcript in each sample was standardized to the amount of PBGD cDNA as the internal control. The amounts of TLR2 mRNA are expressed as the multiplicity of these cDNA concentrations in the calibrator. Each sample was determined in triplicate. Data represent mean ± SD and are representative of groups of six animals in an experiment. *P < 0.05, compared with the control value derived from uninfected mice (Student’s *t* test)

**Fig. 2:**
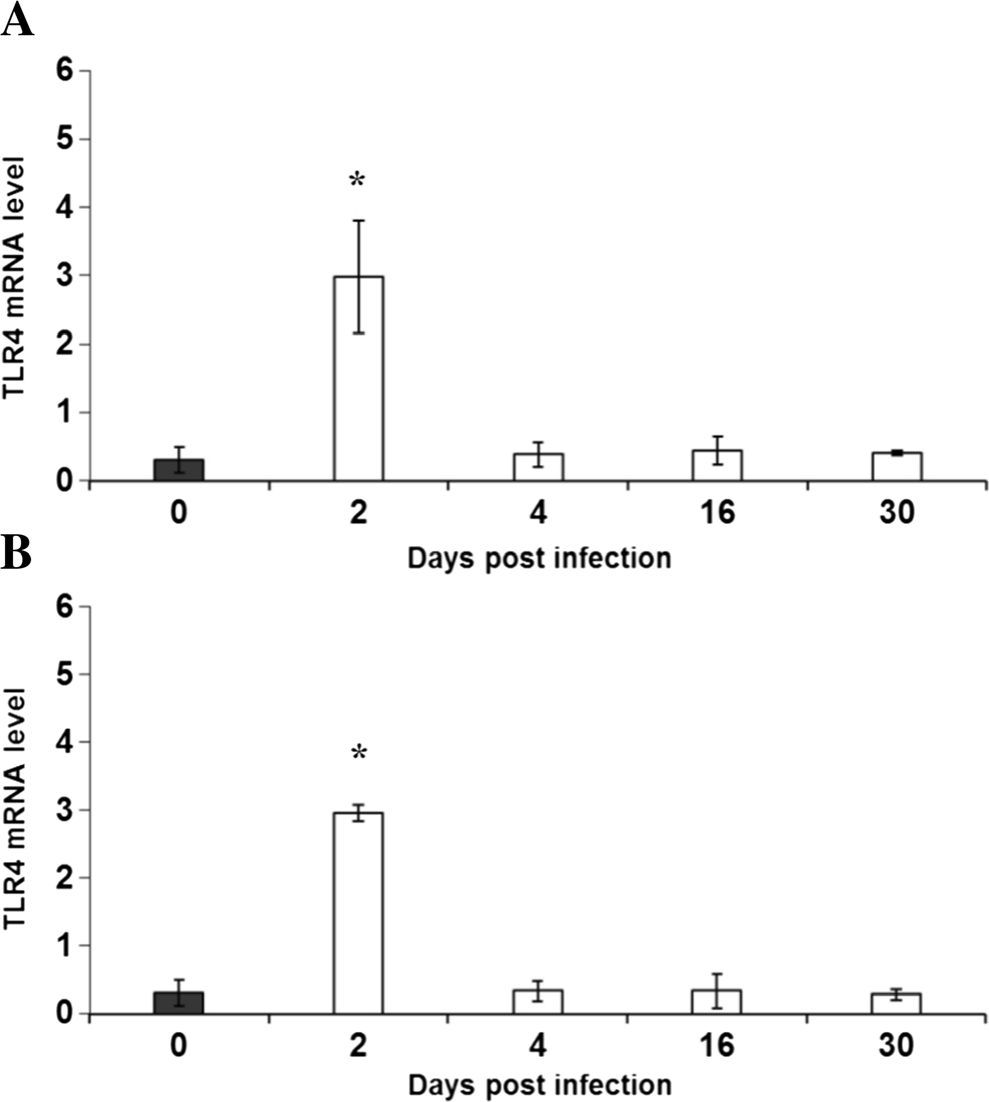
Expression of TLR4 gene at the mRNA level in brains isolated from uninfected and *Acanthamoeba*-infected mice from patient with *Acanthamoeba* keratitis (strain Ac55; **a**) and Malta Lake (strain of Ac43; **b**). Brains were dissected from mice at 2, 4, 16, and 30 dpi. Expression level of TLR4 gene was determined by Q-PCR relative quantification analysis evaluated using a calibrator (cDNA mix from all samples). The quantify of TLR4 transcript in each sample was standardized to the amount of PBGD cDNA as the internal control. The amounts of TLR4 mRNA are expressed as the multiplicity of these cDNA concentrations in the calibrator. Each sample was determined in triplicate. Data represent mean ± SD and are representative of groups of six animals in an experiment. *P < 0.05, compared with the control value derived from uninfected mice (Student’s *t* test)

This study showed that the levels of mRNA expression of Toll-like receptor (TLR2 and TLR4) genes in the control group (uninfected mice) were very similar (Figs. 1 and 2).

In the brain of mice infected by *Acanthamoeba* strains of Ac55 and Ac43, it was observed that the level of mRNA expression of TLR2 statistically increased only at 2 dpi, and at 4 dpi, it was higher but without statistical significance, whereas at 16 and 30 dpi, it was at a similar level compared with the control group (Fig. 1a, b).

The levels of mRNA expression of TLR4 in the brains from the infected mice statistically increased only at 2 dpi, whereas at 4, 16, and 30 dpi, it was at a similar level compared with uninfected mice (Fig. 2a, b).

In the brains of mice infected by *Acanthamoeba* spp. isolated from a patient with *Acanthamoeba* keratitis (Ac55) and Malta Lake (Ac43), the levels of expression of TLR2 were statistically higher than the levels of expression of TLR4.

### Immunohistochemical staining

**Fig. 3:**
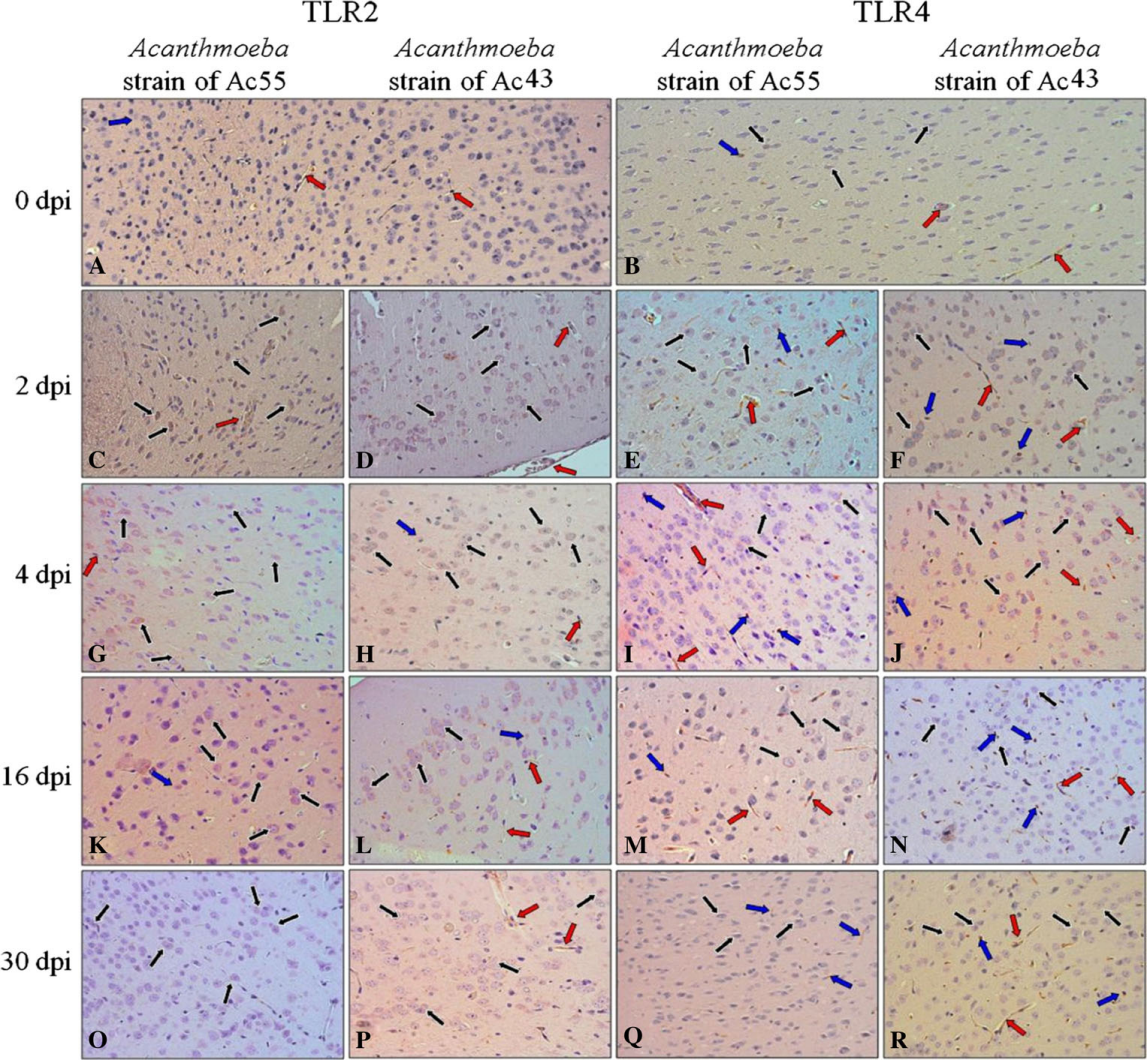
Immunoexpression of Toll-like receptor 2 (TLR2) (**a**, **c**, **d**, **g**, **h**, **k**, **l**, **o**, **p**) and Toll-like receptor 4 (TLR4) (**b**, **e**, **f**, **i**, **j**, **m**, **n**, **q**, **r**) within neocortex of control (**a**, **b**) and mice infected with *Acanthamoeba* spp. isolated from patient with *Acanthamoeba* keratitis strain Ac55 (**c**, **e**, **g**, **i**, **k**, **m**, **o**, **q**) and from Malta Lake strain Ac43 (**d**, **f**, **h**, **j**, **l**, **n**, **p**, **r**) in 2, 4, 16, and 30 dpi. Exemplary immunopositive cells: neurons—*black arrows*; glial cells—*blue arrows*; endothelial cells of neural capillaries—*red arrows*. Intensity of IHC reaction was highest in the 2-dpi group and decreased during the period of infection. Objective magnification ×40

The results of the immunohistochemical reactions, presented in Fig. 3c, d, g, h, k, l, o, p, show that brains (neocortex) of mice infected with *Acanthamoeba* exhibited changes in TLR2 and TLR4 (Fig. 3e, f, i, j, m, n, q, r) intensity in comparison to the control group (Fig. 3a, b).

In control mice brains, both Toll-like receptors were expressed in epithelium of neural blood vessels (Fig. 3a, b; red arrows); TLR2 was sporadically observed in neurons and glial cells (black and blue arrows, respectively). In these groups, TLR4 expression was slightly more intensive than TLR2.

In the neocortex of mice infected by *Acanthamoeba* strain Ac55, TLR2 (Fig. 3c, g, k, l, o) was located mainly in neurons (black arrows); sporadically glial cells (blue arrow) and also infrequently endothelial cells of capillaries (red arrow) were low TLR2-positive. The TLR2 immunoexpression was most intense at 2 and 4 dpi (Fig. 3c, g), and the immunointensity decreased during the time of infection. At 16 and 30 dpi (Fig. 3k, o), the level of TLR2 expression was quite similar to the control group (Fig. 3a) but appeared to be lower.

A brown pigmentation indicated that TLR4 immunohistochemical staining within the neocortex of brains of mice infected by *Acanthamoeba* strain Ac55 (Fig. 3e, i, m, q) was the highest at 2 dpi (Fig. 3e) and markedly decreased during the time of infection (Fig. 3i, m, q). The immunoexpression was observed in neurons (black arrows), glial cells (blue arrows), and capillaries (red arrows). The number of TLR4-positive cells (neurons, glial, and endothelial) was higher than in TLR2 immunostaining experiment.

In the neocortex of mice infected by *Acanthamoeba* strain Ac43, TLR2 (3, D, H, L, P) was located mainly in neurons (blue arrows) and sometimes in glial cells (blue arrows) and endothelial cells (red arrows). The highest expression was noted at 2 dpi (Fig. 3d), lower at 4 dpi (Fig. 3h), and lower, similar to the control, at 16 and 30 dpi (Fig. 3l, p).

TLR4 expression in neocortex of mice infected by *Acanthamoeba* strain Ac43 (Fig. 3f, j, n, r) was analogous to TLR4 expression within the group of mice infected by *Acanthamoeba* strain Ac55. The highest expression was observed at 2 dpi (Fig. 3f) and was much lower at the subsequent days post-infection (Fig. 3j, n, r). Neurons (black arrows) and glial (blue arrows) and endothelial (red arrows) cells were immunopositive.

**Fig. 4:**
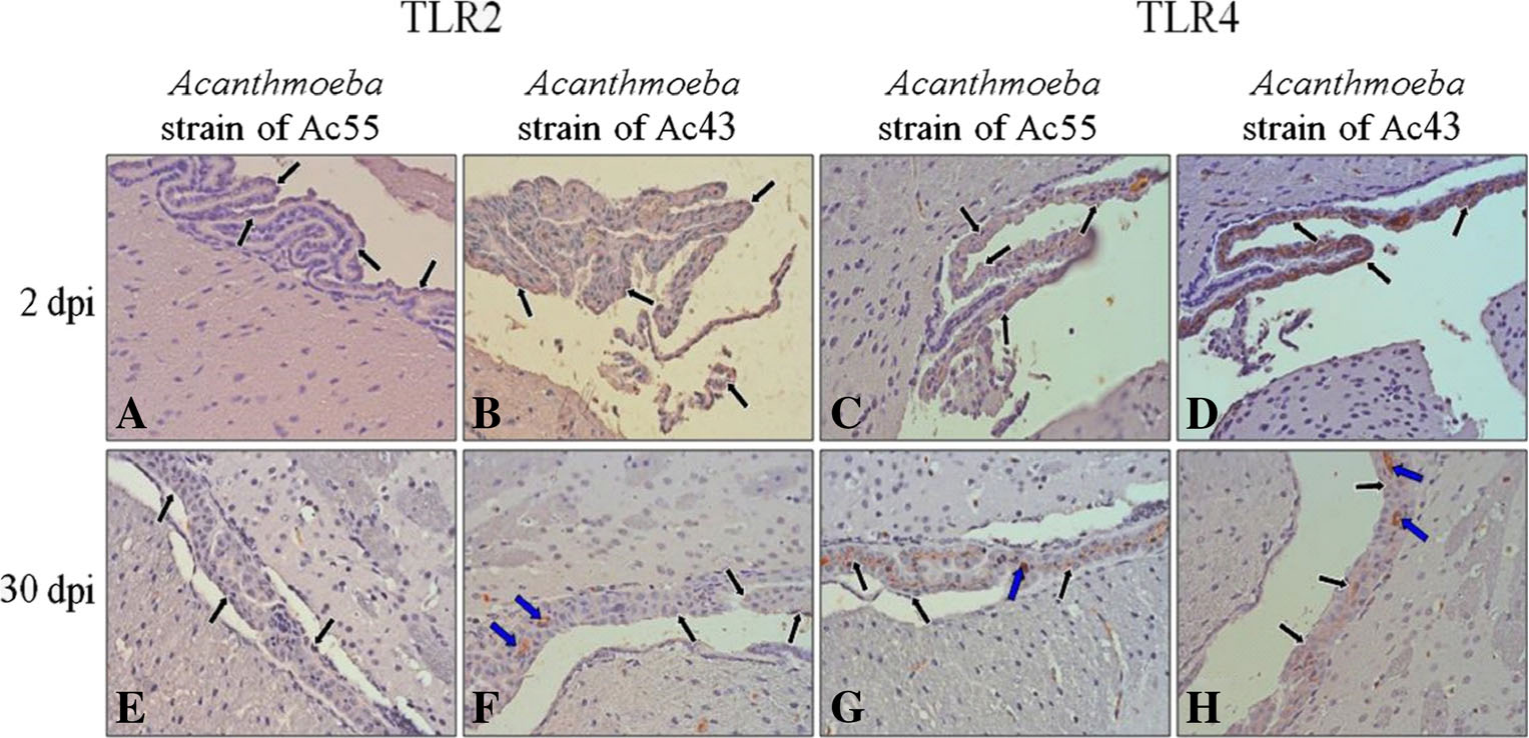
Immunoexpression of Toll-like receptor 2 (TLR2) (**a**, **b**, **e**, **f**) and Toll-like receptor 4 (TLR4) (**c**, **d**, **g**, **h**) within choroid plexus of mice infected with *Acanthamoeba* spp. isolated from patient with *Acanthamoeba* keratitis (strain Ac55) (**a**, **c**, **e**, **g**) and from Malta Lake (strain Ac43) (**b**, **d**, **f**, **h**) at 2 and 30 dpi. Choroidal ependymocytes—*black arrows*; interstitial cells (possibly dendritic cells)—*blue arrows*. Intensity of IHC reaction was the highest in 2 dpi groups and very low in 30 dpi groups. Objective magnification ×40

The changes of immunoexpression of Toll-like receptors were also observed in ependymocytes of the choroid plexus (Fig. 4a–h, black arrows). The highest TLR2 and TLR4 expression levels were at 2 dpi (Fig. 4a–d), decreasing during the time of infection and reaching a minimum at 30 dpi (Fig. 4e–h). TLR expression was much more intense in choroidal ependymocytes of mice infected by *Acanthamoeba* strain Ac43 (Fig. 4b, d, f, h) than Ac55 (Fig. 4a, c, e, g). During the period of infection, TLR expression in ependymocytes fell, but in connective tissue of the choroid plexus, there appeared immunopositive cells (Fig. 4f, g, h; blue arrows), possibly dendritic cells.

## Discussion

The results of this study indicate a neurotropic character of both strains of *Acanthamoeba* (Ac43 and Ac55). In all the infected mice, *Acanthamoeba* spp. were confirmed in the brain. Also, Kasprzak et al. (1974) indicate the brain as the primary site of infection by intranasal inoculation. The most common microscopic changes in the brain include blood effusion resulting from damage to the capillary walls (Rucka 1974; Gieryng and Gieryng 1987; Górnik et al. 2005). Górnik et al. (2005) found, in parts of the meninges and perivascular space of mice infected with *Acanthamoeba* spp., trophozoites of *Acanthamoeba* as well as neutrophils, macrophages, plasma cells, and single multinucleate giant cells.

This study reports the first documentation of the expression of TLR2 and TLR4 mRNA and protein in the brains of *Acanthamoeba* spp.-infected mice. The CNS is an immunologically unique organ because of the presence of the blood–brain barrier (BBB) and the absence of a classically defined lymphatic drainage system (Mishra et al. 2006). Parasitic infection of the CNS (such as malaria, African trypanosomiasis, neurocysticercosis, and amoebic encephalitis) is a major cause of mortality worldwide, second to HIV infection (Mishra et al. 2009). During infection, cells of the CNS have the ability to produce inflammatory mediators such as chemokines, adhesion molecules and cytokines, and costimulatory molecules during infection (Takeda et al. 2001; Dabbagh & Lewis 2003; Chavarria & Alcocer-Varela 2004). In the brain, TLRs, including TLR2 and TLR4, are expressed on microglia, astrocytes, and oligodendrocytes (Bsibsi et al. 2002a, 2002b; Bowman et al. 2003; Olson & Miller 2004). However, in neurons, TLR2 and TLR4 are expressed (Tang et al. 2007). The TLR family of proteins plays an important role in host innate immunity (Hoebe et al. 2004). Once engaged, signaling through TLRs starts from the Toll/interleukin-1 receptor (TIR) domain (Medzhitov 2001) and involves one of four adaptor protein: myeloid differentiation factor 88 (MyD88), MyD88-adaptor-like/TIR-associated proteins (MAL/TIRAP), Toll-receptor-associated activator of interferon (TRIF), and Toll-receptor-associated molecule (TRAM) (Mishra et al. 2009). Moreover, it has been proposed that TLRs control the switch from the innate to the adaptive immune response (Yarovinsky et al. 2005).

In this study, we observed a statistically increased level of expression of TLR2 as well as TLR4 mRNA at 2 dpi in the brains of mice infected with two different strains of *Acanthamoeba*. In *Acanthamoeba-*infected mice, TLR2 and TLR4 expression was detected in neurons, glial cells, and endothelial cells of the neocortex. It is also interesting that TLR2 and TLR4 were more intensively expressed in ependymocytes of the choroid plexus of infected mice at 2 dpi.

Amin et al. (2012) reported that TLR2/9-MyD88-mediated signaling participates in intracerebral control of parasite load in the brain of *T. brucei*-infected mice (Amin et al. 2012). Moreover, Bafica et al. (2006) found that the same TLRs (2 and 9) cooperate in the control of infections by an intracellular parasite, such as *T. cruzi*. However, TLR2 and 9 but not TLR4, 5, and 7 were involved in cerebral malaria (CM) infection using *Plasmodium berghei* ANKA (PbA) (Coban et al. 2006). In contrast with the above results, Lepenies et al. (2008) demonstrated that the induction of CM is independent of TLR2, 4, and 9 caused by *P. berghei* ANKA infection. Moreover, human malaria is associated with higher expression levels of TLRs 1, 2, 4, and 8 and reduced levels of TLRs 3 and 5 (Ockenhouse et al. 2006; Loharungsikul et al. 2008). Additionally, other results suggested that TLR1, 2, 4, 6, and 9 are not independently essential for control of *T. gondii* infection. This result is in contrast with a study finding that TLR2 plays a role in the protective immunity against *T. gondii* infection in the lungs, but its protective function in this organ remains to be clarified (Mun et al. 2003). Importantly, Hitziger et al. (2005) suggested that different results may result from different strains, dose, and route of administration. Particularly, TLR2 is not an essential molecule for protective immunity to low-dose infection, but TLR2 is an essential molecule for protective immunity to high-dose infection of *T. gondii* (300 cysts or more) (Mun et al. 2003). A further study showed that TLR11−/− and TLR2/4 double knockout mice display relatively increased susceptibility to infection with a simultaneous decrease in IL-12 along with an increase in the number of brain cysts (Debierre-Grockiego et al. 2007; Yarovinsky 2008). It is worth noting that tachyzoite heat shock proteins and other partially purified tachyzoite preparations activate TLR4 and TLR2 (Aosai et al. 2002; Del Rio et al. 2004). Recently, a study found that TLR4 might be involved in inflammatory reactions of brain injury to chronic *T. gondii* infection of rats (Zhou et al. 2012). Another study, which involved a comprehensive analysis of TLR expression in the normal and parasite infected brain in a mouse model of neurocysticercosis (*Mesocestoides corti*), suggested a role for TLRs in the interplay of immune cells and CNS cells during infection. Above study indicated that TLRs were differentially distributed among various CNS cell types upon infection, e.g., TLR2 was localized to nervous tissue cells, particularly astrocytes, but TLR4 was localized to microglia and neurons (Mishra et al. 2006). Additionally, among all TLRs, TLR2 expression was induced first and was substantially upregulated in the brain during murine neurocysticercosis (Mishra et al. 2009). Moreover, the results of Gundra et al. (2011) demonstrated that TLR2-mediated responses help to mitigate not only CNS pathology but also mortality due to infection in murine NCC.

In conclusion, the alternative in the level of expression of TLR2 and TLR4 may imply the role of the innate immune system during parasitic infection. A family of proteins called TLRs plays an important role in the induction of inflammatory cytokines during infection, such as by parasites. Increased levels of TLR2 and TLR4 mRNA expression in infected mice suggested the involvement of these TLRs in the recognition of *Acanthamoeba* PAMPs.
